# Theranostic Toolbox for Neutrophil Functionalization

**DOI:** 10.1002/advs.202504412

**Published:** 2025-06-30

**Authors:** Pascal Bouvain, Kay‐Matthias Thomy, Anika Maria Prinz, Bodo Steckel, Shiwa Kadir, Alexandra Röhs, Jonas Schmitz, Claudia Dohle, Matthias Karg, Maria Grandoch, Ulrich Flögel, Sebastian Temme

**Affiliations:** ^1^ Experimental Cardiovascular Imaging Molecular Cardiology Heinrich Heine University 40225 Düsseldorf Germany; ^2^ Institute for Translational Pharmacology Heinrich Heine University 40225 Düsseldorf Germany; ^3^ Institute of Physical Chemistry 1 Heinrich Heine University 40225 Düsseldorf Germany; ^4^ Department of Anesthesiology University Hospital Düsseldorf 40225 Düsseldorf Germany; ^5^ Institute of Chemistry Physical Chemistry of Functional Polymers Martin Luther University Halle‐Wittenberg 06120 Halle (Saale) Germany; ^6^ Cardiovascular Research Institute Düsseldorf (CARID) Heinrich Heine University Düsseldorf Germany

**Keywords:** fluorine, inflammation, MRI, neutrophils, targeted nanoparticles, theranostics

## Abstract

Neutrophils are crucial for fighting invading pathogens and for the clearance of sterile wounds. However, in cases of overwhelming pathogens or excessive inflammatory responses, fine‐tuning of neutrophil functions can help to either enhance bacterial killing or prevent unwanted tissue damage. Thus, the present study is aimed at developing a nanoparticle‐based toolbox to direct neutrophils toward either a proinflammatory or reparative phenotype tailored to the specific inflammatory environment. For this, neutrophil‐specific fluorine nanoparticles additionally equipped with either activating or inhibiting peptides for a background‐free ^19^F MRI‐based theranostic approach are engineered. It is demonstrated that with this i) distinct effector functions can be specifically modulated in both murine and human neutrophils, ii) the disease‐specific degree of neutrophil infiltration can be non‐invasively monitored in vivo, and in turn iii) neutrophil effector functions can either be attenuated during a pharmacological challenge or amplified to ameliorate tissue damage during bacteria‐driven acute colitis. Thus, this nanotheranostic approach is suitable to visualize and concomitantly direct neutrophil functionality depending on the specific requirements to improve disease course and outcome, thus paving the way for a personalized neutrophil immunotherapy.

## Graphical abstract

Here, neutrophil‐specific fluorine nanoparticles additionally equipped with either activating or inhibiting peptides for a background‐free ^19^F MRI‐based nanotheranostic approach are engineered. It is demonstrated that this approach is suitable to visualize and concomitantly direct neutrophil functionality depending on the specific requirements to improve disease course and outcome, thus paving the way for a personalized neutrophil immunotherapy.

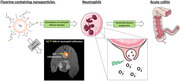

## 1 Introduction

Neutrophils are among the first cells that enter an inflammatory lesion where they exhibit multiple effector functions such as phagocytosis, formation of neutrophil extracellular traps (NETosis), the release of cytokines, enzymes and reactive oxygen species (ROS). Altogether, these effector functions aim to destroy invading pathogens, but are also important to remove cellular debris and to clean up the inflammatory lesion. Moreover, in recent years it has been revealed that neutrophils do also play sophisticated roles in the modulation of adaptive immune responses, stimulate angiogenesis and promote wound healing,^[^ ^1^ ^]^ for example by fostering the differentiation of macrophages into an M2 phenotype. However, an overshooting immune response with excessive neutrophil infiltration is often linked with severe tissue damage and worse outcome of the disease.^[^ ^2^, ^3^, ^4^, ^5^, ^6^ ^]^ In particular, massive ROS secretion and NETosis can damage surrounding tissue and can have detrimental impact on healthy cells and non‐infected tissue^[^ ^2^, ^3^, ^4^ ^]^ Therefore, both the number and functionality of the invading neutrophils are of key relevance to mount an appropriate acute inflammatory reaction that proceeds to the resolution and healing phase without induction of severe tissue damage. Taken together, this illustrates the need to i) monitor neutrophils during initiation and progression of the disease and if necessary ii) to modulate their effector functions for therapeutic purposes (theranostics).

In the past few decades, nanoparticles have been intensively used as drug carriers for theranostics, since they reduce undesirable toxicity and protect the drugs against degradation.^[^ ^7^, ^8^ ^]^ For cell tracking, optical techniques offer excellent sensitivity, but do not provide the required penetration depth for human translation. More suitable for this are non‐invasive whole‐body imaging techniques like computed tomography (CT), positron emission tomography (PET) or magnetic resonance imaging (MRI).^[^ ^9^, ^10^, ^11^ ^]^ However, for visualization of individual cell populations, these have to be loaded with agents which create contrast against the surrounding anatomical structures. Here, PET has been vastly used as very sensitive imaging modality,^[^ ^12^, ^13^ ^]^ but it requires radioactive nuclides with a short half‐life, which prevents long‐term investigation of labeled cells. Furthermore, for accurate assignment of the PET tracer to anatomical structures it is often combined with CT, which exposes the patient – in particular when repetitive investigations are required – to significant amounts of ionizing radiation.

This drawback can conveniently be overcome by MRI, that does not involve harmful radiation and allows the use of the stable flourine‐19 (^19^F) nucleus as cell tracer in combination with conventional hydrogen (^1^H) MR images for precise anatomical location. Of note, ^19^F offers high sensitivity and is nearly absent from biological tissue. To generate ^19^F‐based MRI contrast agents, emulsified tracers or polymers with a high payload of ^19^F are preferentially used.^[^ ^14^, ^15^ ^]^ After intravenous injection, “neat” tracers are readily taken up by phagocytic immune cells, which has been exploited to track their infiltration into inflamed tissue.^[^ ^16^, ^17^, ^18^, ^19^, ^20^, ^21^, ^22^, ^23^ ^]^ However, for selective theranostic approaches toward individual cell types an active targeting of the tracer to these cell populations is required.

**Figure 1:**
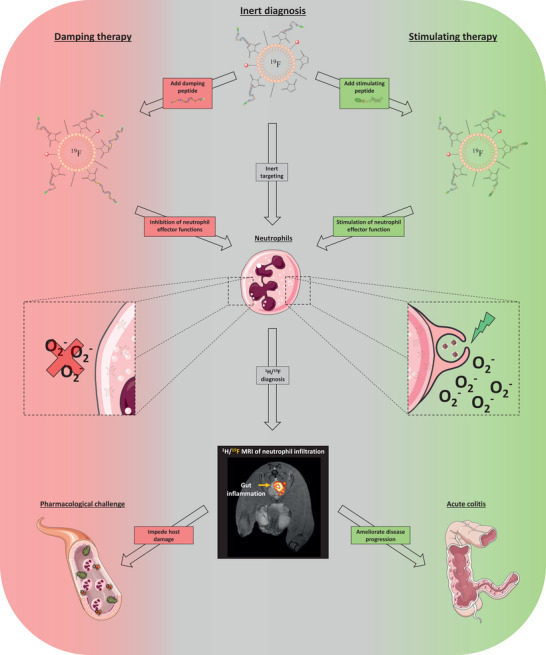
Schematic drawing of the approach used in the present study. Physiologically inert neutrophil‐specific FNPs were conjugated with additional peptides leading to neutrophil‐specific inhibition (left part, red) or activation (right part, green). This modular neutrophil theranostics can be used to boost pathogen destruction (non‐sterile inflammation) or avoid unwanted self‐damage (sterile inflammation) depending on the disease state.

In the present study, we made us of recently reported neutrophil‐specific peptides (NPs) to target both human and murine neutrophils.^[^ ^24^ ^]^ Coupling of these peptides to fluorine‐containing nanoparticles (FNPs) enabled the specific tracking of neutrophils by in vivo ^19^F MRI, but without interference of their functional state. Here, we have taken this approach to the next level by expanding this immunologically “inert” targeting procedure for diagnostic purposes into a highly specific immunomodulatory theranostic toolbox. The current design of the nanoparticles – PEGylation as stealth cap and NP as neutral binding peptide^[^ ^24^ ^]^ – allowed for an absolutely flexible modular approach to steer the therapy in a cell type‐specific manner in any desired direction by adding inhibitory or stimulatory ligands. In a first step, we further improved the targeting efficacy of the neutrophil‐specific FNPs (^NP^FNPs) by optimizing their physicochemical properties. We then functionalized them with immunomodulatory peptides for parallel in vivo visualization and stimulation/inhibition of the neutrophil's effector functions. With this, we provide a nanotechnology platform to concomitantly monitor and direct neutrophils into either a proinflammatory or reparative phenotype tailored to the distinct inflammatory environment (**Figure** **1**), paving the way for personalized neutrophil immunotherapy.

## 2 Results and Discussion

### 2.1 Tuning ^NP^FNPs for Enhanced Neutrophil Targeting

In a first step, we optimized the labelling efficiency of the neutrophil‐specific ^NP^FNPs. Since there is evidence, that increasing particle size leads to higher endocytic uptake,^[^ ^25^ ^]^ we used a 10‐fold reduction of the lipid content during the manufacturing process to generate bigger ^NP^FNPs (^bNP^FNPs) with a hydrodynamic diameter of ≈350 nm compared to the previously described^[^ ^24^ ^]^ which exhibited a substantially smaller diameter of ≈150 nm (^sNP^FNPs) (Figure S1A, Supporting Information). For control experiments, a scrambled, non‐specific peptide (con) was conjugated to both small and big FNPs to generate ^scon^FNPs and ^bcon^FNPs. Characterization of the formulations revealed a slightly increased polydispersion index (PDI), a similar ζ potential and both a somewhat higher fluorine content as well as fluorescence signal for ^bNP^FNPs compared to ^sNP^FNPs (Figure S1A, Supporting Information). Importantly, neutrophil specificity was unaltered by particle size, as exposure of murine blood immune cells to ^bNP^FNPs or ^sNP^FNPs resulted in a 100% labeling of neutrophils for both targeting formulations, while no uptake of the control emulsions or by other immune cell types (lymphocytes or monocytes) occurred (Figure S1B, Supporting Information).

**Figure 2:**
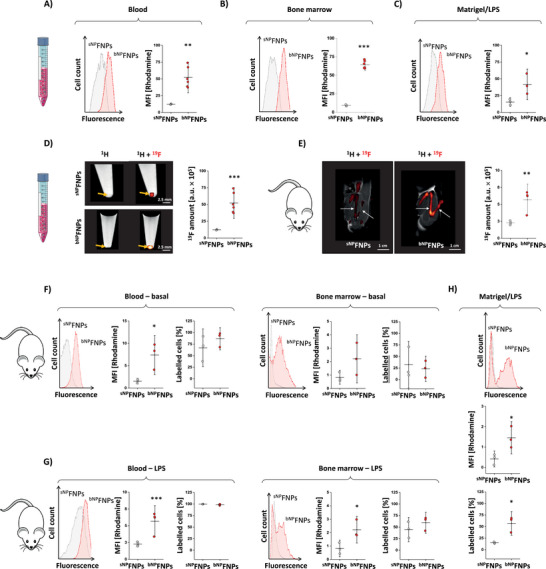
Increased size of ^NP^FNPs leads to a more efficient neutrophil targeting. Neutrophils isolated from the blood, A) the bone marrow B) as well as from subcutaneously implanted matrigel plugs doped with LPS C) were incubated ex vivo with ^sNP^FNPs (grey) or ^bNP^FNPs (red) for 30 min. Afterwards, cellular uptake of ^sNP^FNPs/^bNP^FNPs by neutrophils was determined by flow cytometry. On the left, histogram overlays are shown and quantification of the data (mean fluorescence intensity (MFI)) on the right. D) To investigate ^sNP^FNP/^bNP^FNP labelling of neutrophils by ^19^F MRI, bone marrow neutrophils were isolated, incubated ex vivo with ^sNP^FNPs or ^bNP^FNPs, carefully washed, pelleted by centrifugation and analysed by ^1^H/^19^F MRI. The images display merged ^1^H/^19^F MRI images of buffer‐filled reaction tubes with cells at the bottom. Here and in the following, the ^19^F signal is overlaid in red/orange on the grey‐scale ^1^H MR image. Right: Quantification of the total ^19^F signal intensity. E) ^sNP^FNPs or ^bNP^FNPs were injected intravenously into the tail vein of mice and 24 h later the in vivo labelling of neutrophils in the bone marrow was determined. Ilium, femur and tibia were visualized by T2‐weighted MRI, followed by ^19^F MRI from the same field of view. F) ^sNP^FNPs (grey) or ^bNP^FNPs (red) were intravenously injected and subsequently neutrophils were isolated from the blood and the bone marrow. The cellular uptake of the FNPs (MFI and the number of positive cells) were determined by flow cytometry. G) Matrigel doped with LPS was subcutaneously implanted into the neck of mice to induce a local inflammation. After 24 h ^sNP^FNPs or ^bNP^FNPs were injected intravenously and 1 h later neutrophils from the blood and bone marrow were isolated to determine the FNP uptake by flow cytometry. Left: Histogram overlays of the FNP‐labelled neutrophils; right: Quantification of the relative number of positive cells (middle row) and the mean fluorescence intensity (right row). H) Matrigel/LPS was subcutaneously implanted and excised after 24 h for isolation of neutrophils and analysis of FNP uptake by flow cytometry. Top: Histogram overlay of neutrophils incubated with ^sNP^FNPs (grey) or ^bNP^FNPs (red). Middle and lower panel: Quantitative analysis of the relative number of positive cells (middle) and the mean fluorescence intensity (lower panel). All data sets are mean values ± SD of n = 3–6 (A), n = 3–6 (B), n = 3 (C), n = 5–6 (D), n = 4 (E), n = 3 (F), n = 3 (G) and n = 3 (H). * = *p* < 0.05; ** = *p* < 0.01; *** = *p* < 0.001 verified by Student's t‐test.

To verify the labeling efficiency of ^sNP^FNPs/^bNP^FNPs, murine neutrophils were isolated either i) from the blood pool or ii) the bone marrow and additionally iii) from an inflammatory hot spot which was induced by s.c. implantation of an matrigel plug doped with lipopolysaccharide (LPS) into the neck.^[^ ^26^ ^]^ Ex vivo incubation with ^sNP^FNPs and ^bNP^FNPs, respectively, showed a strongly increased neutrophil labeling for the big particles in all cases – of note, also for activated neutrophils obtained from an inflammatory environment (**Figure** **2**A–C). In separate experiments, we confirmed these findings on the MRI level: Again, neutrophils were isolated from the bone marrow, exposed to ^sNP^FNPs or ^bNP^FNPs and subsequently analyzed by ^1^H/^19^F MRI. Quantitative analysis demonstrated a substantially stronger fluorine incorporation into neutrophils incubated with ^bNP^FNPs compared to ^sNP^FNPs (Figure 2D, ^1^H grey‐scale, ^19^F hot‐iron). For in vivo confirmation, ^sNP^FNPs or ^bNP^FNPs were injected intravenously and after 24 h ^19^F accumulation was determined within the hematopoietic bone marrow niche. Acquisition of spatially corresponding ^1^H/^19^F MRI identified the labeled neutrophils within the bone marrow of femur, tibia, and ilium as ^19^F hot spots and quantification revealed a significant stronger ^19^F signal in the long bones for ^bNP^FNPs compared to ^sNP^FNPs (Figure 2E, ^1^H greyscale, ^19^F hot‐iron).

### 2.2 Strongly Increased In Vivo Labelling of Neutrophils by ^bNP^FNPs without Functional Impact

To resolve the in vivo uptake characteristics of both FNP formulations in more detail, neutrophils were isolated from blood and bone marrow 1 h after ^bNP^FNPs/^sNP^FNPs injection. Similar to the ex vivo findings above (Figure 2A–C), flow cytometry revealed a much stronger uptake by blood neutrophils and also a slight increase in the number of labelled neutrophils upon ^bNP^FNP injection (Figure 2F, left). Corresponding results were obtained for bone marrow neutrophils with largely increased uptake for ^bNP^FNPs, but a lower relative number of labelled cells – most likely due to the short time period after injection limiting the supply to and uptake by the bone marrow (Figure 2F, right). Analogous experiments after activation of neutrophils by matrigel/LPS plug implantation confirmed these findings, but showed a substantially higher number of labelled neutrophils in both the blood and bone marrow (Figure 2G,H). Interestingly, neutrophils isolated from the inflammatory hot spot (matrigel/LPS plug) displayed a three‐times higher uptake of ^bNP^FNP per cell and a three‐fold increased percentage of labeled neutrophils (Figure 2H). Overall, ^bNP^FNPs led to a much stronger cellular incorporation by neutrophils as ^sNP^FNPs which is most likely due to the higher avidity of the bigger particles which means that both more binding ligands and cell surface receptors are engaged which enhances the uptake.

To exclude functional alterations of neutrophils by the increased ^bNP^FNP uptake, we determined expression levels of the early activation marker CD11b upon incubation with ^bNP^FNPs/^sNP^FNPs by flow cytometry. While LPS stimulation as positive control led to significantly increased CD11b surface expression, ^bNP^FNP treatment did not affect neutrophil CD11b expression patterns (Figure S1C, Supporting Information). Furthermore, chemokine‐induced migration was also unaltered under ^bNP^FNP/^sNP^FNP exposure (Figure S1D, Supporting Information) as was neutrophil viability over time (Figure S1E, Supporting Information). Given the superior targeting efficiency of the bigger particles without any side effects, we next aimed to modify ^bNP^FNPs to specifically regulate the functionality of neutrophils by additional conjugation of activating/inhibiting peptides.

### 2.3 Generation and Characterization of Neutrophil Activating ^aNP^FNPs

**Figure 3:**
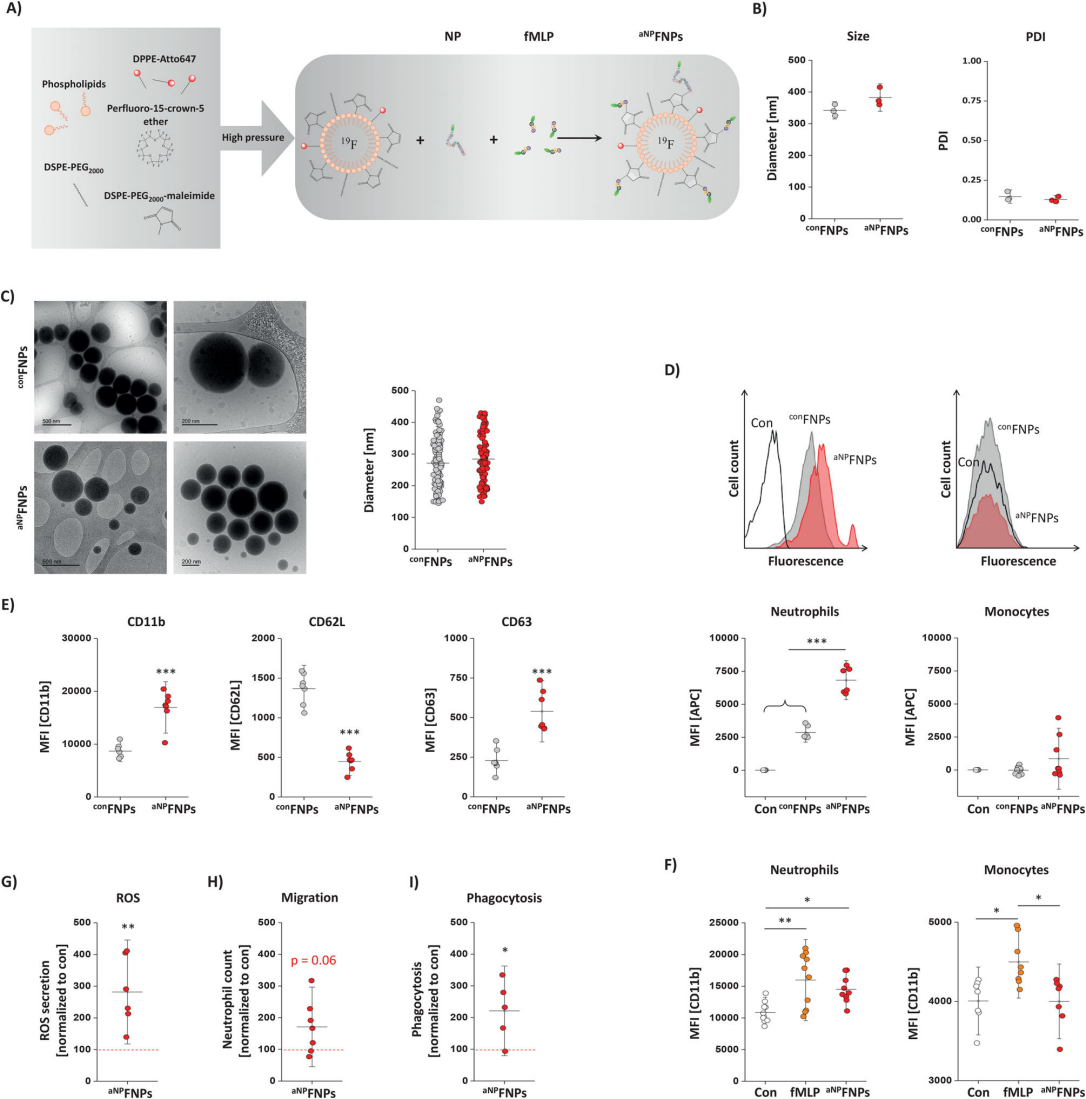
Generation and characterization of neutrophil activating nanoparticles (^aNP^FNPs). A) Schematic overview of the FNP preparation that contain both the peptide NP to target murine neutrophils and the activating ligand fMLP (N‐formylmethionine‐leucyl‐phenylalanine) termed ^aNP^FNPs. As control FNPs equipped with NP only were used (^con^FNPs). (B) Characterization of ^con^FNPs and ^aNP^FNPs regarding their size and PDI via DLS. C) Left: Cryo‐TEM of ^con^FNPs and ^aNP^FNPs. FNPs appear as dark electron dense, round shaped structures. Right: Quantitative analysis of the FNP diameter from the images on the left. D) Murine blood immune cells were incubated with ^con^FNPs (grey) and ^aNP^FNPs (red) followed by staining against CD11b, Ly6G and Ly6c and flow cytometric analysis to determine the cellular uptake of the FNPs (MFI = mean fluorescence intensity) by neutrophils (CD11b^+^, Ly6G^+^) and monocytes (CD11b^+^, L6G^‐^). Con = cells incubated without FNPs (white). Upper panel: Histogram overlay of neutrophils (left) and monocytes (right) incubated with FNPs. Lower panel: Quantitative analysis of the MFI. E) Isolated murine blood immune cells were incubated with ^con^FNPs or ^aNP^FNPs and the cell surface expression of the activation markers CD11b, CD62L and CD63 was analyzed by flow cytometry. F) CD11b cell surface expression on neutrophils and monocytes after exposure to ^aNP^FNPs (red) or free fMLP (orange). Untreated cells served as control (Con, white). G) ROS secretion by neutrophils upon ^aNP^FNP incubation was determined by treatment of the supernatant with dihydroethidium (DHE) followed by ultra‐performance liquid chromatography (UPLC) H) Migration of isolated neutrophils incubated with ^aNP^FNP or ^con^FNP toward fMLP determined in a Boyden chamber. I) ex vivo phagocytic uptake of FITC‐labelled *E coli* by neutrophils. Uptake was determined by flow cytometry. G‐I: The quantitative analyses show the relative increase in DHE oxidation, migration and *E. coli* labelling in cells treated with ^aNP^FNPs compared to ^con^FNPs. All data sets are mean values ± SD of n = 3 (B), n = 1 (C), n = 6 – 10 (D, F), n = 6–7 (E), n = 6 (G), n = 7 (H) and n = 5 (I). * = *p* < 0.05; ** = *p* < 0.01; *** = *p* < 0.001 verified by one‐way ANOVA (D, F) or Student's t‐test (E, G, H, I).

To this end, we first made use of N‐formylmethionine‐leucyl‐phenylalanine (fMLP) which is well known to bind with high affinity to the formyl peptide receptor (FPR1) and leads to cell activation by G‐protein‐coupled signal transduction pathways.^[^ ^27^ ^]^ For conjugation of fMLP to ^bNP^FNPs, the peptide was modified with a terminal cysteine for click chemistry with the maleimide groups on the FNP surface to form activating ^aNP^FNPs (see **Figure** **3**A for a schematic overview). As control, non‐fMLP‐ but NP‐equipped big targeting particles were used, termed here as ^con^FNPs. Physicochemical characterization with dynamic light scattering (DLS) demonstrated a similar size of ≈350 nm for ^aNP^FNPs/^con^FNPs and a PDI of roughly 0.13, indicating a homogenous size distribution (Figure 3B). In cryo‐transmission electron microscopy (cryo‐TEM), electron‐dense FNPs appear as dark, round structures (Figure 3C) with, importantly, no lucent liposomes visible in both types of FNP preparations. Quantification of ^aNP^FNP/^con^FNP diameters in cryo‐TEM images revealed a similar size of ≈300 nm (Figure 3C) comparable to the DLS measurements. Small differences are expectable since DLS determines the hydrodynamic diameter and cryo‐TEM the droplet diameter.

Since the fMLP‐receptor FPR1 is not exclusively expressed by neutrophils, but can also be found on monocytes and macrophages,^[^ ^28^ ^]^ we next investigated the targeting specificity of ^aNP^FNPs and incubated immune cells isolated from the blood of healthy mice with ^aNP^FNPs and ^con^FNPs, respectively. Subsequent flow cytometry revealed that the high binding specificity of the targeted ^con^FNPs for neutrophils was even doubled by additional fMLP conjugation (Figure 3D, left), while for monocytes the minor binding of ^con^FNPs was only slightly increased by ^aNP^FNPs (Figure 3D, right). To verify in more detail that ^aNP^FNPs indeed lead to a stimulation of neutrophil effector functions, we incubated isolated neutrophils with ^aNP^FNPs/^con^FNPs and subsequently determined surface expression of the activation markers CD11b, CD62L and CD63 by flow cytometry (Figure 3E). While expression of CD11b and CD63 was more than doubled in neutrophils exposed to ^aNP^FNPs, the surface expression of CD62L dropped by a factor of three, indicating that adding fMLP to ^bNP^FNPs not only leads to an enhanced binding but also a strong activation of murine neutrophils. On the other hand, only the co‐conjugation of fMLP with NP on the surface of the targeting FNPs ensures the neutrophil specificity of this chemokine: While exposure to “neat” fMLP led to a similar upregulation of the early activation marker CD11b in both monocytes and neutrophils (Figure 3F), ^aNP^FNPs did not at all impact on CD11b expression of monocytes as compared to control conditions, but resulted in almost equivalent activation of neutrophils as the free cytokine (Figure 3F). Obviously, NP on the surface directs the particles predominantly to neutrophils, so that the co‐bound fMLP also mainly acts on this cell type.

### 2.4 ^aNP^FNPs Enhance Neutrophil Effector Functions and Improve Bacterial Clearance In Vivo

On the functional level, we found in parallel ROS secretion by neutrophils to be nearly tripled (Figure 3G), migration was almost doubled (Figure 3H) and phagocytosis of *E. coli* (Figure 3I) was also more than doubled after ^aNP^FNP treatment (always in comparison to ^con^FNPs). In translational experiments, we found similar stimulatory effects of ^aNP^FNPs and free fMLP also for human neutrophils and monocytes (Figures S2 and S3, Supporting Information).

**Figure 4:**
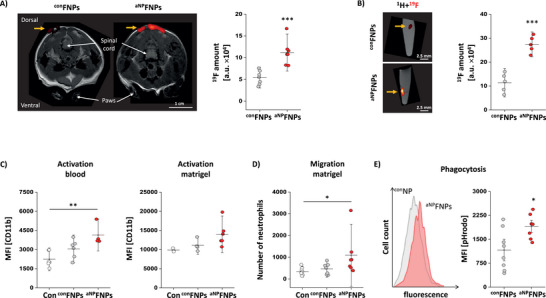
^aNP^FNPs increase neutrophil effector functions in vivo. A) Matrigel/LPS plugs were subcutaneously implanted into the neck of mice followed by injection of ^con^FNPs or ^aNP^FNPs 30 min later. After 24 h, in vivo MRI measurements were conducted. Left: ^1^H/^19^F MR images of the matrigel area in axial orientation. Yellow arrows indicate the location of the matrigel plug. Right: Quantitative analysis of the total ^19^F signal intensity from the matrigel area. B) ex vivo ^1^H/^19^F MRI of matrigel plugs excised 24 h after injection of FNPs. Left: Sagittal ^1^H/^19^F MR images of reaction tubes with excised matrigel plugs (arrow) and filled with PBS. Right: Quantification of the total ^19^F signal. C) Left: Matrigel/LPS implantation was followed by intravenous ^con^FNPs/^aNP^FNPs injection and after 1 h immune cells were isolated from the blood for determination of CD11b cell surface expression on neutrophils by flow cytometry (left). Right: One day after matrigel/LPS implantation ^con^FNPs/^aNP^FNPs were i.v. injected. Matrigels were excised further 24 h later and cell surface levels of CD11b were analysed on neutrophils infiltrated into plug. Con = cells from mice that did not receive any FNPs. D) As functional readout, migration of neutrophils into the matrigel/LPS plug was determined by flow cytometry in parallel to ^19^F MRI analysis. ^aNP^FNPs were injected i.v. and 1 h later matrigel/LPS was implanted into the neck of mice. Further 2 h later, the matrigel‐plug was excised and the number of infiltrated neutrophils was assessed. E) As phagocytosis assay, mice received an intravenous injection of ^aNP^FNPs and 30 min later fluorescent *E. Coli* particles were i.v. injected. After additional 30 min, immune cells from the blood were isolated and the uptake of the *E. Coli* particles by neutrophils was determined via flow cytometry. On the left, a histogram overlay of the *E. Coli* uptake (grey = ^con^FNPs; red = ^aNP^FNPs) is displayed while quantification on the right shows a significant stronger uptake of *E. coli* by ^aNP^FNPs‐treated animals. All data sets are mean value ± SD of n = 7 (A), n = 3–6 (B), n = 4 – 6 (C), n = 7 (D) and n = 8 (E). * = *p* < 0.05; ** = *p* < 0.01; *** = *p* < 0.001 verified by One‐way ANOVA (C, D) or Student's t‐test (A, B, E).

Next, we examined the suitability of ^aNP^FNPs for tracking and activation of neutrophils in an inflammatory environment in vivo. Local inflammation was again induced by implantation of a matrigel/LPS plug into the neck of mice followed by intravenous injection of ^aNP^FNPs/^con^FNPs. After 24 hrs, ^1^H/^19^F MRI revealed substantially increased ^19^F signals within the LPS‐doped matrigel (yellow arrows) for ^aNP^FNPs compared to ^con^FNPs (**Figure** **4**A). These in vivo findings were subsequently confirmed by ex vivo ^1^H/^19^F MRI after plug explantation (Figure 4B). At the same time, ^aNP^FNPs resulted in increased CD11b expression on neutrophils isolated from the blood and matrigel: Already 1 h after application of ^aNP^FNPs, CD11b expression was significantly higher in circulating neutrophils as compared to saline‐treated control animals (Con) whereas ^con^FNPs did not result in a pronounced upregulation (Figure 4C, left); similar effects of ^aNP^FNPs were detectable for neutrophils isolated from the matrigel (Figure 4C, right). Additional experiments demonstrated that the upregulation of CD11b by ^aNP^FNPs led also to an increased infiltration of neutrophils into the inflammatory hot spot (Figure 4D).

In a separate scenario, we investigated whether ^aNP^FNPs could as well amplify the antimicrobial effector functions of neutrophils. To this end, ^aNP^FNPs/^con^FNPs were applied intravenously 30 min prior to an *E. Coli* injection into the tail vein. Further 30 min later, immune cells were isolated from the blood and phagocytosis of the pHRodo‐labeled bacteria by neutrophils was quantified by flow cytometry. As shown in Figure 4E, pre‐stimulation of neutrophils by ^aNP^FNPs indeed led to a more efficient phagocytosis of *E. Coli* as compared to ^con^FNPs further corroborating the therapeutic potential of our approach.

### 2.5 Application of ^aNP^PFCs Improves the Outcome in Acute DSS‐Induced Colitis

**Figure 5:**
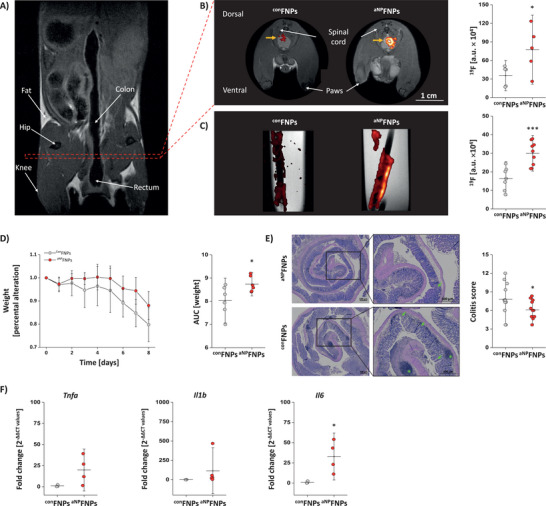
Improved outcome in experimental colitis by ^aNP^FNP‐enhanced neutrophil activation. Mice were treated with dextran sodium sulphate (DSS) for 8 days via the tap water. At distinct time points (days 0, 1, 4, 7) mice received i.v. injections of ^con^FNPs or ^aNP^FNPs. On day 8, the abdominal area and the gut were analyzed by ^1^H/^19^F MRI. A) Coronal view of the area interest. The red rectangle indicates the location of the axial image plane in B). B) Left: Axial ^1^H/^19^F MR images of the lower abdomen in the area of the colon from DSS‐treated mice that received ^con^FNPs or ^aNP^FNPs. Right: Quantification of the total ^19^F signal intensity in the colon. C) Left: ex vivo sagittal ^1^H/^19^F MR images of dissected guts derived from DSS‐treated mice that received ^con^FNPs/^aNP^FNPs. The quantitative analysis of the corresponding ^19^F content is shown on the right. D) Relative body weight (normalized to the baseline weight on day 0 of the animals over the period of the DSS treatment. E) Hematoxylin and eosin staining of 8 µm sections of the gut of DSS‐treated mice that received ^aNP^FNPs (upper) or ^con^FNPs (lower). Middle: Magnified areas were asterisks indicate lymphatic follicles and arrows deformed crypts. Right: Quantification of the colitis tissue injury score based on morphological features. F) qPCR analysis (fold change normalized to controls) of the expression levels of TNFα, IL1β and IL6 in gut tissue samples from DSS‐treated mice that received ^con^FNPs/^aNP^FNPs. All data sets are mean value ± SD of n = 5 (B), n = 7–9 (C), n = 5–6 (D), n = 11–12 (E) and n = 4–5 (F). * = *p* < 0.05; *** = *p* < 0.001 verified by Student's t‐test.

Based on the above findings that ^aNP^FNPs led to a more efficient clearance of bacteria from the blood, we next explored their theranostic suitability in a murine colitis model well known to be driven by massive bacterial infiltration.^[^ ^29^ ^]^ Here, exposure to dextran sodium sulfate (DSS) via the drinking water leads to damaging of the gut wall that triggers severe inflammation of the intestinal wall. Thus, we hypothesized that boosting neutrophil effector functions in the early phase of the disease might help to attenuate bacterial invasion and lower gut inflammation as well as subsequent tissue damage. To this end, mice were treated with ^aNP^FNPs/^con^FNPs immediately prior as well as on day 1, 4 and 7 after initiation of DSS exposure. In vivo ^1^H/^19^F MRI on day 8 revealed a significantly increased neutrophil‐specific ^19^F signal in the gut for ^aNP^FNP‐ compared to ^con^FNP‐treated animals (**Figure** **5**A,B). These findings were validated by ex vivo ^1^H/^19^F MRI of the gut, confirming substantial larger ^19^F intensities after ^aNP^FNP versus ^con^FNP treatment (Figure 5C). Importantly, ^aNP^FNP therapy led also to a significantly ameliorated weight loss of mice under DSS exposure over the entire observation period (Figure 5D). The notion of an improved outcome by the ^aNP^FNP‐induced activation of neutrophils was further corroborated by the overall histologic colitis score considering among others the appearance and number of lymphatic follicles as well as deformed crypts, which both were significantly decreased by ^aNP^FNPs (Figure 5E). Further investigation of the gut revealed upregulated activation markers (*Tnfa*, *Il1b*, *Il6*) for ^aNP^FNP‐treated animals underlining the more enhanced neutrophil effector functions in these mice (Figure 5F). Overall, the data indicate that ^aNP^FNP‐mediated neutrophil stimulation can indeed support the host response against invading bacteria and ameliorate disease progression in the present colitis model.

### 2.6 Neutrophil Inhibiting ^iNP^FNPs Damp Neutrophil Function In Vitro and In Vivo

Since neutrophil activation is a double‐edged sword as many diseases are related to hyperactivated neutrophils, e. g. excessive ROS secretion or NETosis,^[^ ^2^, ^3^, ^4^, ^5^, ^6^ ^]^ we tried to extend our approach to also attenuate their effector functions. For this, we made use of the peptide EPICC which has been reported to modulate myeloperoxidase activity resulting in less release of ROS.^[^ ^30^ ^]^ In a first step, we confirmed reduced secretion of ROS under EPICC exposure in both isolated murine and human neutrophils as determined by UPLC (Figure S4A,B, Supporting Information).

**Figure 6:**
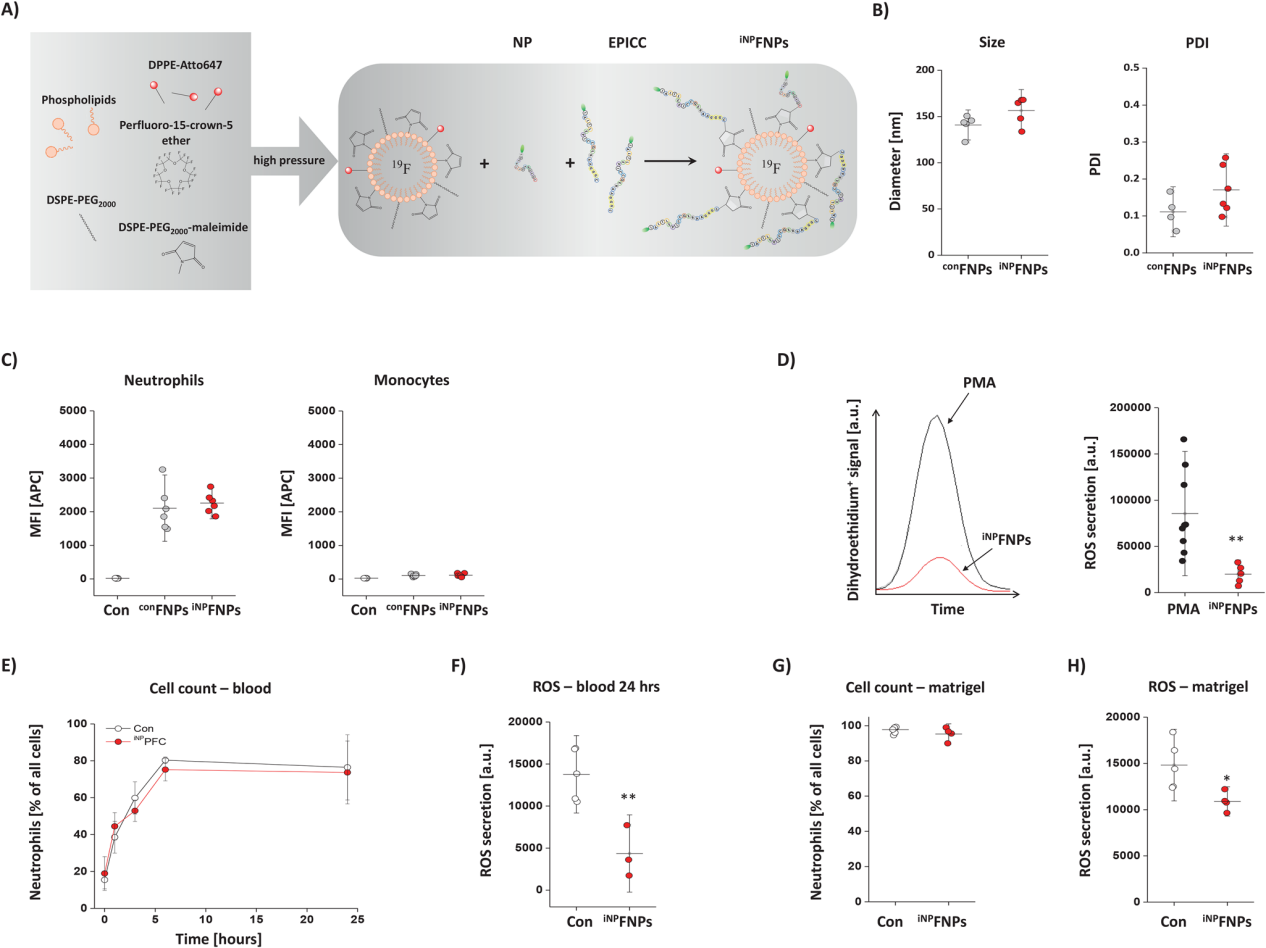
Formation and characterization of neutrophil inhibiting nanoparticles (^iNP^FNPs). A) Schematic overview of the manufacturing process to generate FNPs that contain both the peptide NP to target murine neutrophils and the inhibiting peptide EPICC, termed ^iNP^FNPs. As control FNPs equipped with NP only were used (^con^FNPs) B) Characterization of ^con^FNPs and ^iNP^FNPs regarding their size and PDI via DLS. C) Isolated murine blood immune cells were incubated with ^con^FNPs and ^iNP^FNPs for 30 min followed by flow cytometric analysis. As negative control cells did not receive any FNPs (Con). D) To verify functionality of ^iNP^FNPs, isolated murine neutrophils were stimulated in HBSS buffer with PMA to induce ROS secretion as well as 10 µM DHE for assessment of extracellular ROS. In parallel, neutrophils were incubated with ^iNP^FNPs. After 60 min, the supernatant was analysed by UPLC (right). On the left a representative histogram is given for the different treatments. E–H) Mice received a matrigel/LPS plug to induce local inflammation followed by i.v. ^iNP^FNP injection. E) At distinct time points (0, 1, 3, 6, and 24 h) blood samples were withdrawn from the tail vein. Subsequently, immune cells were isolated and analyzed by flow cytometry to quantify circulating neutrophils. F) ROS assay with neutrophils isolated from the blood 24 h after plug implantation and ^iNP^FNP injection in comparison to animals treated with matrigel/LPS and saline (Con). G) The matrigel plug was excised 24 h after implantation and neutrophil content was determined by flow cytometry. H) ROS production of neutrophils isolated from the matrigel after ^iNP^FNP treatment (or injection of saline as control). All data sets are mean value ± SD of n = 3 (B), n = 6 (C), n = 9 (D), n = 9 (E), n = 4 (F), n = 4 (G), and n = 4 (H). ** = *p* < 0.01; *** = *p* < 0.001 verified by Student's t‐test.

Next, we coupled EPICC to ^NP^FNPs for preparation of functionalized ^iNP^FNPs (**Figure** **6**A). Physicochemical characterization revealed no significant differences between ^iNP^FNPs or corresponding ^con^FNPs with regard to particle diameter or PDI (Figure 6B). Furthermore, both targeting efficacy and specificity were not affected by additional coupling of EPICC: No difference was observed between ^iNP^FNP and ^con^FNP binding to neutrophils and no unspecific uptake by monocytes occurred (Figure 6C). Furthermore, no other effector functions like phagocytosis or migration were found to be impaired for murine neutrophils (Figure S4C, Supporting Information). To explore whether ^iNP^FNPs could be indeed used to specifically damp neutrophil ROS secretion, ROS levels were determined after stimulation of isolated neutrophils with phorbol myristate acetate (PMA). As shown in Figure 6D, co‐incubation with ^iNP^FNPs significantly reduced the PMA‐induced ROS production in comparison to solely PMA‐treated neutrophils. In separate experiments, also human neutrophils were exposed to ^iNP^FNPs, which resulted in similar inhibitory effects on PMA‐induced ROS production (Figure S4D, Supporting Information). Of note, free EPICC was required for both species in mM concentrations for detectable effects (Figure S4A,B, Supporting Information). However, estimation of the EPICC content in ^iNP^FNPs yielded a maximum value of ≈100 µM, thus an order of magnitude lower, but with even stronger effects on ROS inhibition in both murine and human neutrophils (Figure 6D; Figure S4D, Supporting Information). Again, it seems that co‐conjugation of the active compound with NP to the particle surface leads to a more potent effect on the target cell type, which might help to overcome potential side effects.

Finally, we verified the functionality of ^iNP^FNPs also in vivo making again use of the matrigel/LPS model to induce a defined local inflammation. At distinct time points after plug implantation, blood samples were withdrawn for determination of circulating neutrophil numbers, their recruitment into the inflammatory hot spot and their ROS secretion. As expected, implantation of the LPS‐doped matrigel increased neutrophil content in the blood from roughly 20% up to 80% within 24 h (Figure 6E). While this rise in circulating neutrophils was unaffected by ^iNP^FNPs injected in parallel to the matrigel implantation, at the same time one of their main effector functions was almost completely blunted by ^iNP^FNP treatment in that ROS secretion was diminished to a third of the control group (Figure 6F). Analysis of the explanted matrigel plug after 24 h resulted in similar findings with a comparable number of recruited neutrophils in both groups (Figure 6G), but a significantly attenuated ROS secretion by neutrophils isolated from the inflammatory hot spot when ^iNP^FNP were applied (Figure 6H). Taken together, these results indicate that ^NP^FNPs can also successfully modified to substantially reduce ROS production by neutrophils to damp an excessive immune response and to avoid unwanted damage of viable tissue in an inflammatory environment.

## 3 Conclusions

Taken together, our approach allows the parallel visualization and modulation of neutrophil effector functions to support the body fighting infectious agents or to decrease an overshooting immune response to foster resolution and healing. Especially in elderly persons the function of neutrophils is often impaired which is accompanied by augmented infections, a process which is called immune senescence.^[^ ^31^ ^]^ Thus, targeted neutrophil activation by ^aNP^FNPs might be an option to overcome an impaired immunity in the elderly. Of course, stimulation strength must be tightly surveilled to avoid hyperactivation, but this might be realized by monitoring neutrophil dynamics by ^19^F MRI. On the other hand, we provide evidence that also theranostic approaches to attenuate neutrophil function can be realized using ^iNP^FNPs keeping neutrophils in an inactivated state.

In summary, we demonstrate the successful engineering of specific FNPs to boost sensitivity for in vivo tracking of neutrophils by ^1^H/^19^F MRI and that this can be further exploited as a platform for theranostic functionalization of neutrophils either in a stimulating or damping manner – tailored to the respective inflammatory environment. The translational potential of this technology is huge as fluorine coils can readily be interfaced with clinical scanners, simple “neat” FNPs have already explored in clinical trails^[^ ^32^, ^33^ ^]^ and the feasibility of their detection by in vivo ^19^F MRI has previously been demonstrated in the clinical setting.^[^ ^34^, ^35^ ^]^ Importantly, since our modular approach is not limited to FNPs and MRI, the used peptides can easily be conjugated to tracers for other imaging modalities. Furthermore, other disease entities, target epitopes or cell types could be approached by the same technology assuming the design of suitable ligands, e.g. against monocytes, T or B cells rendering the presented approach as a general and versatile theranostic platform.

## 4 Experimental Section

### Preparation and Characterization of Fluorine‐Containing Nanoparticles (FNPs)

*Peptide Synthesis*: Neutrophil peptides used in this study were previously identified by phage display screening approaches.^[^ ^36^, ^37^ ^]^ It was modified all peptides (human/murine NP, Con, fMLP, EPICC) sequences by adding three glycine (‐GGG‐) as spacer, a C‐terminal cysteine for coupling reactions followed by and an N‐terminal carboxyfluorescein to enable detection by fluorescence microscopy and flow cytometry. Peptides were synthesized by Genaxxon Bioscience GmbH (Ulm, Germany) with a purity of more than 95% as verified by HPLC measurements. Peptide sequences: mNP: DFYKPMPNLRIT‐GGG‐C; corresponding Con: SLAMFLTHSPEP‐GGG‐C; hNP, DLVTSKLQV‐GGG‐C; corresponding Con, KQLSEMVTD‐GGG‐C fMLP: MLF‐GGG‐C; EPICC: IALILEPICCQERAA‐GGG‐C.

*FNP Preparation*: Fluorine‐containing nanoparticles (FNPs) were essentially prepared as previously reported. In brief, all ingredients from Table S1 (Supporting Information) without the perfluoro‐15‐crown‐5‐ether were pre‐emulsified for 30 min. Next, perfluoro‐15‐crown‐5 ether was added to the dispersion and a crude emulsion was formed by high shear mixing (Ultra Turrax TP 18/10; IKA‐Werke, Staufen, Germany). High shear homogenization was performed in 5 cycles at 1000 bar using LV1 microfluidizer (Microfluidics Corp, Westwood, MA, USA).

### Preparation of Targeting FNPs and Theranostic FNPs

For the generation of targeting FNPs (^sNP^FNPs, ^bNP^FNPs or corresponding ^con^FNPs), peptides (human/murine NP or respective Con) were used in 5‐molar shortfall to the available maleimide and linked to the maleimide via the free sulfhydryl group of the C‐terminal cysteine. For preparation of ^aNP^FNPs, NP was coupled first as described above and afterwards fMLP was conjugated to the FNPs in a 1:5 or 1:10 molar shortfall with regard to the total amount of the DSPE‐PEG_2000_‐maleimide. For preparation of ^iNP^FNPs, mNP (0.01 mol%) was coupled first to the emulsion in a 1:5 molar shortfall and afterwards the EPICC was linked to the FNPs (0.04 mol%). After incubation for 24 h at 20 °C the emulsions were stored at −20 °C.

For all ex vivo experiments, cells were incubated with 10 µl FNPs/ml which corresponds to ≈80 mmol ^19^F nuclei per ml. For in vivo experiments, mice received a body weight (BW) adapted injection of FNPs with 3 mmol PFCE/kg BW (→ ≈65 mmol ^19^F nuclei per kg BW) via the tail vein. The applied amount of lipids (with phosphatidylcholine as main component of Lipoid S75 (≈70%)) was ≈0.4 and 0.04 mmol kg^−1^ BW for the small and the big nanoparticles, respectively.

*FNP Characterization*: FNPs were characterized by dynamic light scattering (DLS) on a Nanotrac instrument (Microtrac, Krefeld, Germany) to determine the hydrodynamic diameter, the polydispersity index (PDI) and the ζ potential. To this end, FNPs were diluted 1:100 in H_2_O and measured for 30 s each measurement and 10 repeats. Furthermore, the fluorescence by an IVIS Lumina II imaging system (Perkin Elmer, Rodgau, Germany) was analyzed and the ^19^F amount by MRI as described below. To measure the fluorescence, 10 µl of FNPs were spotted on a glass plate, while analysis of the ^19^F content was conducted with 10 µl FNPs in a PCR tube.

### Cryo‐Transmission Electron Microscopy (Cryo‐TEM

FNP nanoemulsions were characterized by Cryo‐TEM analysis. For this the Lacey TEM grids were clamped into the Cryo Plunger Leica EM GP2. Afterwards 4 µl of sample were applied onto the grid and an automated blotting was performed for 10 s. This was followed by automated plunging into liquid ethane. Afterwards, the grid was manually transferred into liquid nitrogen. The grid was mounted into the precooled Fischione Cryo TEM Grid Sample Holder and inserted into the TEM. Finally, measurements were conducted at 200 kV with a Jeol JEM‐2100Plus. Images were taken with a Gatan OneView 4k camera.

### Animal Experiments

Animal experiments were approved by the “Landesamt für Natur, Umwelt und Verbraucherschutz Nordrhein‐Westfalen” and were performed in accordance with the national guidelines on animal care Az: 81‐02.04.2018.A468 and 81‐02.04.2020.A161. Male mice (C57BL/6; 20–30 g BW (body weight); 10–12 weeks of age) used in this study were obtained from Janvier (Le Genest‐Saint‐Isle, France), housed at the central animal facility of the Heinrich Heine University (Düsseldorf, Germany) and fed with a standard chow diet and received tap water ad libitum. All studies with human samples were conducted after informed consent according to the Declaration of Helsinki and local ethics board approval (Ethikkommission, Universitätsklinikum Düsseldorf, Germany; file references 2017–2947_1). All study participants gave written informed consent.

### Immune Cell Isolation from Human Blood

Blood was collected from the vena brachialis, and erythrocytes were lysed by adding the 4‐fold volume of ammonium chloride buffer (pH 7.4; University Hospital Düsseldorf, Pharmacy). After 10 min of incubation, samples were centrifuged at 350 g for 10 min at 20 °C.

### Immune Cell Isolation from Murine Blood or Bone Marrow

To obtain circulating immune cells, heparinized blood was withdrawn by venous puncture of the inferior vena cava. Blood was collected via a 23G cannula in heparin‐aerated collection tubes. Erythrocytes were lysed by adding the 4‐fold volume of ammonium chloride buffer (pH 7.4). After 10 min of incubation at room temperature the samples were centrifuged at 350 × g for 10 min at 20 °C. For isolation of neutrophils from the bone marrow, mice were sacrificed via cervical dislocation and tibia and femur were dissected. Afterwards, cells were isolated from the bone marrow using a protocol by Amend et al.^[^ ^38^ ^]^ To obtain a pure neutrophil population the EasySep mouse neutrophil enrichment kit from StemCell was used.

### Matrigel/LPS Experiments

To induce defined inflammatory foci, a recently developed model of localized subcutaneous inflammation was adopted.^[^ ^26^ ^]^ To this end, 50 µl of ice‐cold matrigel (Corning, Berlin, Germany) were doped with 50 µg of LPS (lipopolysaccharide, *Salmonella typhimurium*, Sigma–Aldrich, Missouri, U.S.A) and s. c. implanted into the neck of mice. This model was employed to explore the impact of a local LPS‐dependent inflammatory stimulus on ^sNP^FNP and ^bNP^FNP labeling of neutrophils. Twenty‐four hour after implantation of the plug, neutrophils were isolated from the blood, the bone marrow and the matrigel, incubated ex vivo with 10 µl ml^−1 sNP^FNP or ^bNP^FNP and subsequently analyzed by flow cytometry (see below). In separate experiments, 24 h after plug implantation, 3 mmol kg^−1^ BW ^sNP^FNP or ^bNP^FNP were injected intravenously into mice with matrigel/LPS and immune cells were isolated from the blood after 1 h for analysis by flow cytometry (see below). For ^19^F MRI measurements, mice were transferred into the MRI 24 h after injection of 3 mmol kg^−1^ BW ^aNP^FNPs or ^con^FNPs and the ^19^F signals were detected around the matrigel plug (see below). Afterwards, matrigel plug was carefully dissected and fixed with PFA to determine the fluorine signal within the matrigel via ex vivo ^1^H/^19^F MRI.

### Isolation of Immune Cells from Matrigel/LPS Plugs

Mice were sacrificed by cervical dislocation and the matrigel plug (see below for implantation procedure) was carefully excised. The matrigel was meshed through a cell strainer (40 µm) and isolated cells were resuspended in MACS for further experiments. For ^19^F images, matrigels were fixed with 100 µl PFA for 15 min. and kept in PBS for analysis.

### Cellular Uptake of FNPs by Circulating Immune Cells and Bone Marrow Neutrophils

To assess incorporation of the different emulsions by murine immune cells in vivo, mice were kept in anesthesia by 1.5 vol% isoflurane on a warming plate. Afterwards, 3 mmol kg^−1^ BW ^sNP^FNPs or ^bNP^FNPs were injected intravenously into the tail vein. One hour upon injection, the blood from the vena cava and the bone marrow from the femur was collected and isolated immune cells were analyzed for their FNP incorporation by flow cytometry (see below) or 24 h after injection the ^19^F incorporation was determined via ^19^F MRI (see below).

### In Vivo Migration of Neutrophils

To investigate the migratory capacities of neutrophils the matrigel/LPS model was used and the number of neutrophils infiltrating the matrigel were determined via flow cytometry. One hour before matrigel implantation 50 µl ^sNP^FNPs or ^bNP^FNPs were injected intravenously. Two hours after matrigel implantation the neutrophils within the matrigel were counted.

### In Vivo Phagocytosis by Neutrophils

To determine the phagocytic properties of neutrophils, mice were treated with 50 µl ^aNP^FNPs or related ^Con^FNPs and 30 min later 50 µl of pHrodo‐labeled *E. Coli* particles were injected intravenously. Again, 30 min later immune cells from the blood were isolated and the phagocytosis of the *E. Coli* particles by neutrophils was determined by flow cytometry.

### Production of Reactive Oxygen Species (ROS)

Three mmol kg^−1^ BW ^iNP^FNPs or saline were injected i. v. followed by implantation of 50 µl matrigel/LPS into the neck. Over time (0, 1, 3, 6, and 24 h) 20 µl blood samples were taken from the tail and the number of neutrophils were determined by flow cytometry. 24 h after injection and implantation, blood and matrigel were isolated and the generation of reactive oxygen species was determined by treatment of the cells with dihydroethidium (DHE).^[^ ^39^ ^]^ For this, 1 × 10^5^ isolated neutrophils were incubated with 20 µM DHE in HBSS buffer (Hanks balanced salt solution, Massachusetts, U.S.A.) for 30 min at 37 °C. After centrifugation, 80 µl of the cell supernatant subjected to UPLC measurement (Waters Acquity Bio H‐Class with 2475 FLD Detector) to determine ROS. To this end, gradients A and B (0.1% trifluoroacetic acid in 1 l water and acetonitrile, respectively) at a flow rate of 0.26 ml min^−1^ at 17 °C were used. DHE and the red fluorescent reaction product was excited and detected at 480 and 580 nm, respectively.

### Induction of Colitis

For induction of colitis mice were treated with 2% DSS (dextran sodium sulfate) via tap water over 8 days. Mice were i. v. injected with 3 mmol kg^−1^ BW ^aNP^FNPs or ^Con^FNPs on day 0, 1, 4 and 7 after the DSS treatment. At day 8 the MRI measurements were performed and afterwards mice were sacrificed, the intestine was isolated, fixed with PFA for 24 h and again measured ex vivo by MRI. Afterwards, histological slices were prepared to determine a colitis score. The weight of the mice was protocolled daily.

### Magnetic Resonance Imaging (MRI)

*General*: All experiments were performed at a vertical 9.4 T Bruker AVANCE^III^ Wide Bore NMR spectrometer (Bruker, Rheinstetten, Germany) operating at frequencies of 400.21 MHz for ^1^H and 376.54 MHz for ^19^F measurements using a Bruker microimaging unit Micro 2.5 with actively shielded gradient sets (1.5 T m^−1^). Data were acquired using a 25 mm quadrature ^19^F resonator with one channel tunable to both ^1^H and ^19^F. Mice were anaesthetized with 1.5% isoflurane and kept at 37 °C. For bone marrow measurements, mice were placed within the resonator in a physiological crouched posture to cover both femur and tibia within the field‐of‐view (FOV). Respiration was monitored by means of a pneumatic pillow positioned at the animal's back and vital function was acquired by a M1025 system (SA Instruments, Stony Brook, NY, USA). After acquisition of morphological ^1^H images, the resonator was tuned to ^19^F and anatomically matching ^19^F images were recorded. For all ^19^F MRI measurements the same reference power and receiver gain were chosen to ensure comparable ^19^F signal intensities between data sets. To fade out the background noise from ^19^F images a constant threshold was applied to ^19^F data and signals originating from the liver were masked for sake of clarity.

*Bone Marrow*: After acquisition of axial pilot scans, two separate slice packages were placed in sagittal orientation to cover the complete bone marrow in tibia and femur in both legs. Scan details: ^1^H rapid acquisition with relaxation enhancement (RARE); repetition time (TR)  =  2000 ms, FOV  =  4.00 × 2.56 cm^2^, matrix: 256 × 256, slice thickness (ST) 1 mm, acquisition time (TAcq) 1 min. ^19^F RARE: TR  =  2500 ms, FOV  =  4.00 × 2.56 cm^2^, matrix: 64 × 64, ST 3 mm, TAcq 10 min.

*Matrigel*: After longitudinal pilot scans the complete matrigel was analyzed by serial images in axial direction. Scan details: ^1^H rapid acquisition with relaxation enhancement (RARE); repetition time (TR)  =  2000 ms, FOV  =  2.56 × 2.56 cm^2^, matrix: 256 × 256, slice thickness (ST) 1 mm, acquisition time (TAcq) 1 min. ^19^F RARE: TR  =  2500 ms, FOV  =  2.56 × 2.56 cm^2^, matrix: 64 × 64, ST 3 mm, TAcq 20 min. In case of ex vivo scans the matrigel was visualized in a longitudinal scan followed by corresponding ^19^F scans. Scan details: ^1^H rapid acquisition with relaxation enhancement (RARE); repetition time (TR)  =  2000 ms, FOV  =  2.56 × 2.56 cm^2^, matrix: 256 × 256, slice thickness (ST) 1 mm, acquisition time (TAcq) 1 min. ^19^F RARE: TR  =  2500 ms, FOV  =  2.56 × 2.56 cm^2^, matrix: 64 × 64, ST 3 mm, TAcq 20 min.

*FNPs*: For evaluation of the ^19^F content, 10 µl of the emulsions were transferred into 200‐µl reaction tubes and measured with the following parameters. ^1^H: RARE, TR  =  3500 ms, RARE factor 16, FOV  =  2.56 × 2.56 cm^2^, matrix: 128 × 128, ST 1 mm, TAcq 1 min. ^19^F: RARE, TR  =  2500 ms, RARE factor 32, FOV  =  2.56 × 2.56 cm^2^, matrix: 32 × 32, ST 1 mm, TAcq 5 min.

*Colitis*: After longitudinal pilot scans, the gut was covered in axial direction over the complete abdomen. Scan details: ^1^H rapid acquisition with relaxation enhancement (RARE); repetition time (TR)  =  2000 ms, FOV  =  2.56 × 2.56 cm^2^, matrix: 256 × 256, slice thickness (ST) 1 mm, acquisition time (TAcq) 1 min. ^19^F RARE: TR  =  2500 ms, FOV  =  2.56 × 2.56 cm^2^, matrix: 64 × 64, ST 3 mm, TAcq 34 min. In case of ex vivo scans the gut was imaged longitudinally followed by corresponding ^19^F scans. Scan details: ^1^H rapid acquisition with relaxation enhancement (RARE); repetition time (TR)  =  2000 ms, FOV  =  2.56 × 2.56 cm^2^, matrix: 256 × 256, slice thickness (ST) 1 mm, acquisition time (TAcq) 1 min. ^19^F RARE: TR  =  2500 ms, FOV  =  2.56 × 2.56 cm^2^, matrix: 64 × 64, ST 3 mm, TAcq 34 min.

*Data Analysis*: MR data were analyzed using in‐house developed software modules based on the LabVIEW package (National Instruments, Austin, TX) as described previously.^[^ ^40^, ^41^ ^]^

### Flow Cytometry

*General*: Flow cytometry was performed with a FACS Canto II (BD Biosciences, USA) or at a LSR Fortessa (BD Biosciences, USA). Cells were gated with appropriate forward/side scatter settings and thresholds for excluding debris. To omit dead cells, samples were stained with 1 µg ml^−1^ DAPI (4′,6‐Diamidin‐2‐phenylindol, Merck). For analysis, cells were gated with FACS Diva software and the mean fluorescence intensities and/or the number of positive cells were determined, depending on the experiment.

*Murine Immune Cells*: The individual mouse immune cell populations were discriminated by antibody staining against CD45 (BD Biosciences, clone 30‐F11), CD11b (Biolegend, clone M1/70) CD11c (Biolegend, clone N418) and Ly6G (BD Biosciences, clone 1A8); lymphocytes: CD45^+^, CD11b^−^, CD11c^−^, Ly6G^−^; classical monocytes: CD45^+^, CD11b^+^, CD11c^+^, Ly6G^−^; non‐classical monocytes: CD45^+^, CD11b^+^, CD11c^−^, Ly6G^−^; neutrophil granulocytes: CD45^+^, CD11b^+^, CD11c^−^, Ly6G^+^. Cells were stained for 20 min at 4 °C, followed by washing with 200 µl MACS buffer. The different emulsions were incubated at a concentration of 10 µl mL^−1^ for the indicated period of time followed by two washing steps with 200 µl MACS buffer.

*Human Immune Cells*: Human immune cells were discriminated by staining for CD45 (BioLegend, clone HI30), CD11b (BD Biosciences, clone ICRF44), CD14 (BioLegend, clone M5E2) and CD16 (BD Biosciences, clone 3G8) (lymphocytes, CD45^+^, CD11b^−^, CD16^−^; classical monocytes, CD45^+^, CD11b^+^, CD14^+^, CD16^−^; non‐classical monocytes, CD45^+^, CD11b^+^, CD14^+^, CD16^+^; neutrophils, CD45^+^, CD11b^+^, CD16^+^).

### Experiments with Cells

*Binding and Internalization of Fluorine Nanoparticles*: To assess binding of Atto647‐labeled ^sNP^FNPs or ^bNP^FNPs to murine immune cells, 1 × 10^6^ cells were incubated with 10 µl ml^−1^ of the FNPs over a period of 30 min at 37 °C under constant shaking in a 1.5 mL tube. After incubation, 50 µl of the samples were transferred into 2 ml ice‐cold MACS buffer to stop the uptake and analyzed for the Atto647 fluorescence by flow cytometry. To determine the ^sNP^FNP/^bNP^FNP uptake by neutrophils via ^19^F MRI, 1 × 10^7^ cells were incubated with 10 µl ml^−1 sNP^FNP/^bNP^FNP in 10 ml DMEM for 4 h. Afterwards, cells were washed several times with 2 mL of PBS transferred into a PCR tube and fixed with 100 µl PFA.

*Cell Surface Activation Marker upon FNP Incubation*: To determine the activation state of neutrophils upon labeling with FNP, cells were incubated with 10 µl ml^−1 sNP^FNPs, ^bNP^FNPs, ^aNP^FNPs or related ^con^FNPs for 30 min at 37 °C under constant shaking. Afterwards, cells were washed twice and the surface expression of early activation markers (CD11b, CD63 and CD62L for mice; CD11b, CD63 and CD66b for human) was determined via flow cytometric analysis. As negative control, cells were incubated in medium only for 30 min. and as positive control neutrophils were activated with 0.1 µg mlc^−1^ LPS or 100 nM fMLP. For concentration dependent activation of neutrophils via fMLP, it was coupled to the ^aNP^FNPs in a 1:5 molar ratio (high dose) or 1:10 molar ratio (low dose).

*Migration of Neutrophils upon FNP Incubation*: Neutrophils were incubated with 10 µl ml^−1 aNP^FNPs or related ^on^FNPs for 30 min at 37 °C followed by intensive washing. Afterwards a migration assay against the chemokine fMLP was performed for 1 h at 37 °C using a Boyden chamber system. The number of migrated neutrophils was determined via flow cytometry. As positive control, neutrophils were pre‐activated with 0.1 µg ml^−1^ LPS for 1 hr.

*E. Coli Phagocytosis by Neutrophils upon FNP Incubation*: 1 × 10^6^ isolated neutrophils were incubated for 30 min with 10 µl ml^−1 aNP^FNPs/^iNP^FNPs in DMEM (Dulbeccos modified eagle medium, Thermo Fisher, Massachusetts, U.S.A.) at 37 °C. Afterwards, cells were washed twice with DMEM and incubated for 30 min with 50 µg mL^−1^ FITC‐labeled *E. coli* particles followed by intensive washing. Uptake of *E. Coli* particles was determined by flow cytometry.

*Viability of Neutrophils upon FNP Incubation*: To investigate the viability of neutrophils upon ^sNP^FNP or ^bNP^FNP incubation, samples were taken after distinct time points (10, 20, 30, 40, 50, and 60 min) of incubation in media at 37 °C. The number of dead neutrophils was determined via DAPI staining and flow cytometry analysis.

*Secretion of Reactive Oxygen Species*: 1 × 10^5^ isolated murine or human neutrophils were incubated for 30 min with 10 µl ^aNP^FNPs, ^iNP^FNPs or related ^con^FNPs or in case of the dose‐response curve with different concentrations of EPICC (1 mM, 10 µM, 100 nM, 1 nM, 10 pM, 100 fM) in 100 µl MACS buffer. Afterwards, cells were washed twice with 200 µl MACS and subsequently incubated with DHE‐HBSS buffer (20 µM) for 60 min at 37 °C. Thereafter, cells were pelleted by centrifugation and 80 µl of the supernatant was used to determine ROS by UPLC measurement (Waters Acquity Bio H‐Class with 2475 FLD Detector). To this end, gradients A and B (0.1% trifluoroacetic acid in 1 l water and acetonitrile, respectively) at a flow rate of 0.26 ml min^−1^ at 17 °C were used. DHE was excited and detected at 480 and 580 nm, respectively. In case of ^iNP^FNPs and the dose‐response curves, 100 nM PMA was added to HBSS‐DHE buffer to induce ROS production.

### Gut Histology and Assessment of Tissue Damage

Five µm sections of paraffin‐embedded whole colon tissue were prepared. For assessment of disease severity using the colitis score sections were stained with hematoxylin and eosin. Parameters and scoring system were listed in Table S2 (Supporting Information) and were adapted from Lahat et al.^[^ ^42^ ^]^ Blinded scoring was carried out by three independent investigators and data were averaged for final quantification.

### Gene Expression Analysis

Total RNA from a maximum of 30 mg frozen colon tissue per sample was isolated using the RNeasy fibrous Tissue Kit (Qiagen, Hilden, Germany) according to the manufacturer's instructions. The RNA was quantified via photometric measurement at 260/280 nm and quality checked using LabChip technology. 1000 ng RNA was reverse transcribed to generate cDNA using Superscript II Reverse Transkriptase Kit (Invitrogen, Carlsbad, USA) according to the manufacturer's instructions. Expression of mRNA levels of *Tnfa*, *Il1b* and *Il6* in the colon was determined by realtime qPCR using 7300 Real Time PCR System (Thermo Fisher Scientific) and Platinum SYBR Green qPCR Super‐Mix‐UDG (Thermo Fisher Scientific). For comparison of relative mRNA expression, the 2^− ΔΔCt^ method was used with 18s as housekeeper gene.

### Microscopy

Microscope pictures of colon histological sections were taken using the digital Leica IMAGER M2 microscope (Leica Microsystems GmbH, Nussloch, Germany) in combination with the Axio Cam HR camera (Carl Zeiss MicroImaging GmbH, Göttingen, Germany) and the Axio Vision 4.9.1.0 software (Carl Zeiss Microscopy GmbH).

### Statistics

No statistical methods were used to predetermine sample size. Experiments were not randomized, and the investigators were not blinded during experiments and outcome assessment. Unless otherwise indicated, all values are given as mean ± SD. Statistical analysis was performed using OriginPro 2016 (OriginLab). Data were tested for Gaussian distribution using the D'Agostino and Pearson omnibus normality test. For comparison of parameters between the groups, a Student's t‐test or One‐ or Two‐way ANOVA was used.

## Conflict of Interest

The authors declare no conflict of interest.

## Author Contributions

U.F. and S.T. contributed equally to this work. P.B., M.G., U.F., and S.T. performed study conception and design; P.B., K.M.T., A.M.P., B.S., S.K., A.R., J.S., and C.D. performed execution of experiments and acquisition of data; P.B., K.M.T., A.M.P., B.S., S.K., M.K., M.G., U.F., and S.T. performed analysis and interpretation of data; P.B. performed recruitment of human blood and tissue samples; P.B. and S.T. performed drafting of manuscript; M.G. and U.F. performed critical revision; M.G., U.F. and S.T. provided funding.

## Supporting information



Supporting Information

## Data Availability

The raw data that support the findings of this study are available from the corresponding author upon reasonable request.
