# Supplementary material for: Theranostic Toolbox for Neutrophil Functionalization
Source: Adv Sci (Weinh). 2025 Jun 30;12(34):e04412. doi: 10.1002/advs.202504412 (PMC12442632; doi:10.1002/advs.202504412)
Supplement: Supplementary file 1 — Supporting Information [file ADVS-12-e04412-s001.pdf]

ADVANCED SCIENCE
Open Access

Supporting Information

for *Adv. Sci.*, DOI 10.1002/advs.202504412

Theranostic Toolbox for Neutrophil Functionalization

Pascal Bouvain, Kay-Matthias Thomy, Anika Maria Prinz, Bodo Steckel, Shiwa Kadir, Alexandra Röhs, Jonas Schmitz, Claudia Dohle, Matthias Karg, Maria Grandoch, Ulrich Flögel and Sebastian Temme*

Figure S1: Characterization of ^{sNP}FNPs/^{bNP}FNPs

A) Ten µl/ml ^{sNP}FNPs (grey) and ^{bNP}FNPs (red) were characterized regarding their size, size distribution and ζ potential by DLS. Furthermore, the fluorine content as well as the fluorescence intensity was determined *via* ^{19}F MRI or fluorescence spectroscopy, respectively. **B)** To evaluate the targeting specificity of ^{sNP}FNPs and ^{bNP}FNPs, murine immune cells were isolated from the blood and incubated for 30 min with the different particles. After washing, the binding of the particles to the different immune cell types (monocytes, lymphocytes and neutrophils) were determined *via* flow cytometric analysis. **C)** To exclude that ^{bNP}FNPs will impact on neutrophil activity, expression of the early activation marker CD11b was analyzed by flow cytometry. For this, neutrophils were incubated alone, with ^{sNP}FNPs, ^{bNP}FNPs or as a positive control with LPS. Histograms on the left show neutrophils after the treatment while quantification on the right display the normalized values of CD11b to the negative control. **D)** Migratory capacities of neutrophils upon ^{bNP}FNPs treatment: Neutrophils were incubated with ^{sNP}FNPs/^{bNP}FNPs or stimulated with LPS prior migration. Afterwards, cells were washed twice and a migration assay carried out. Migrated neutrophils were counted via flow cytometry. **E)**. Neutrophil viability over time during ^{sNP}FNPs/ ^{bNP}FNPs incubation analyzed by flow cytometry and DAPI staining. On the left, exemplarily dot blots are given where DAPI negative and positive cells are shown while the quantification is given on the right. All data sets are mean value ± SD of n =3 (A), n = 3 (B), n = 3 (C), n = 4-5 (D) and n = 3 (E). *** = $p < 0.001$ verified by one-way ANOVA (C).

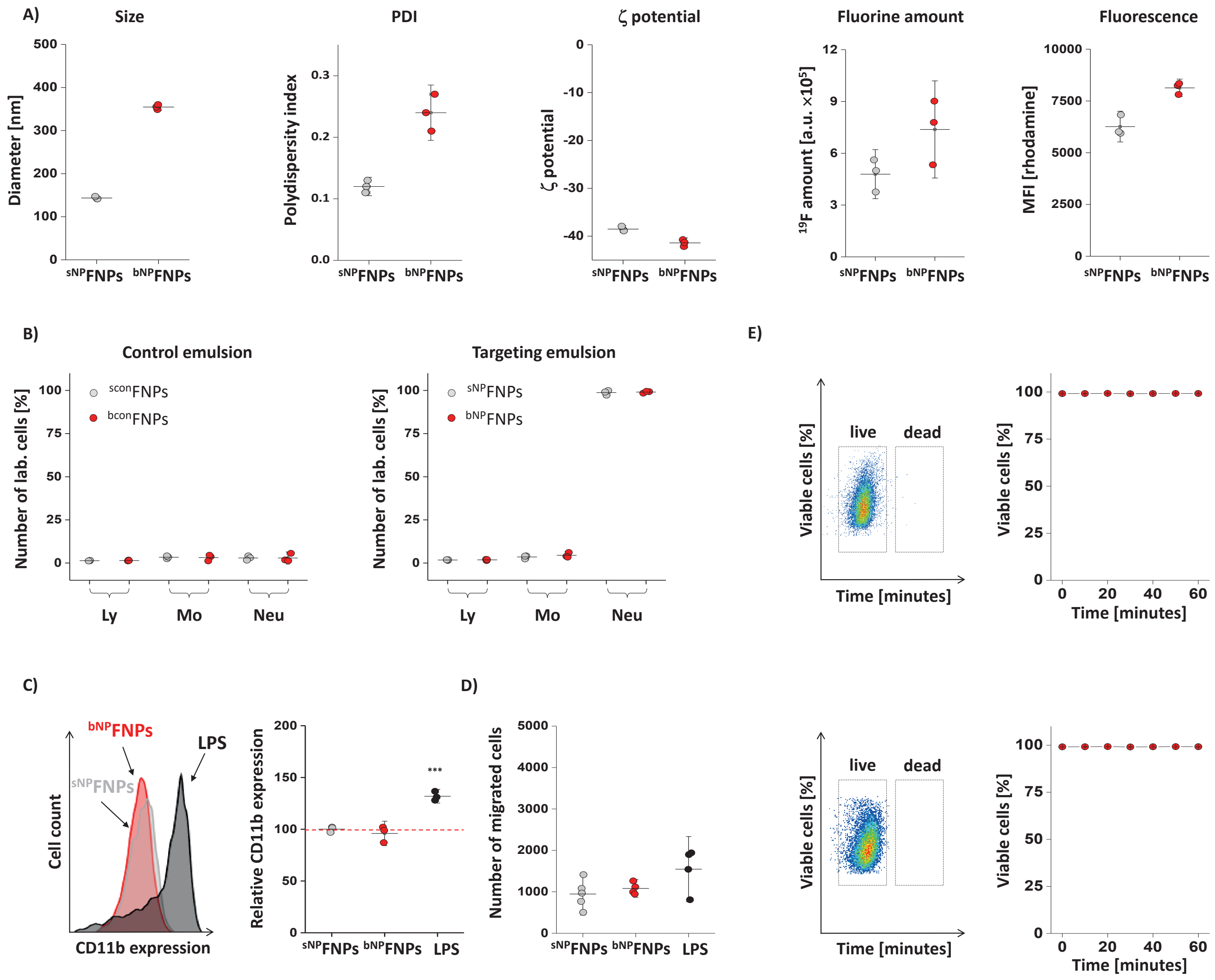

Figure S2: Activation of human neutrophils by ^{aNP}FNPs

Human neutrophils were isolated and incubated with ^{con}FNPs, ^{aNP}FNPs or were left untreated. Afterwards, different surface expressions of early activation markers were determined via flow cytometry. As shown, we identified a significant upregulation of CD63 and CD66b upon ^{aNP}FNPs incubation. Furthermore, the secretion of reactive oxygen species was determined upon ^{con}FNPs/^{aNP}FNPs stimulation via a dihydroethidium assay followed by UPLC measurements. Treatment with ^{aNP}FNPs lead to significant increased secretion of reactive oxygen species compared to untreated or ^{con}FNPs treated neutrophils All data sets are mean value $\pm$ SD of n = 3, verified by one-way ANOVA.

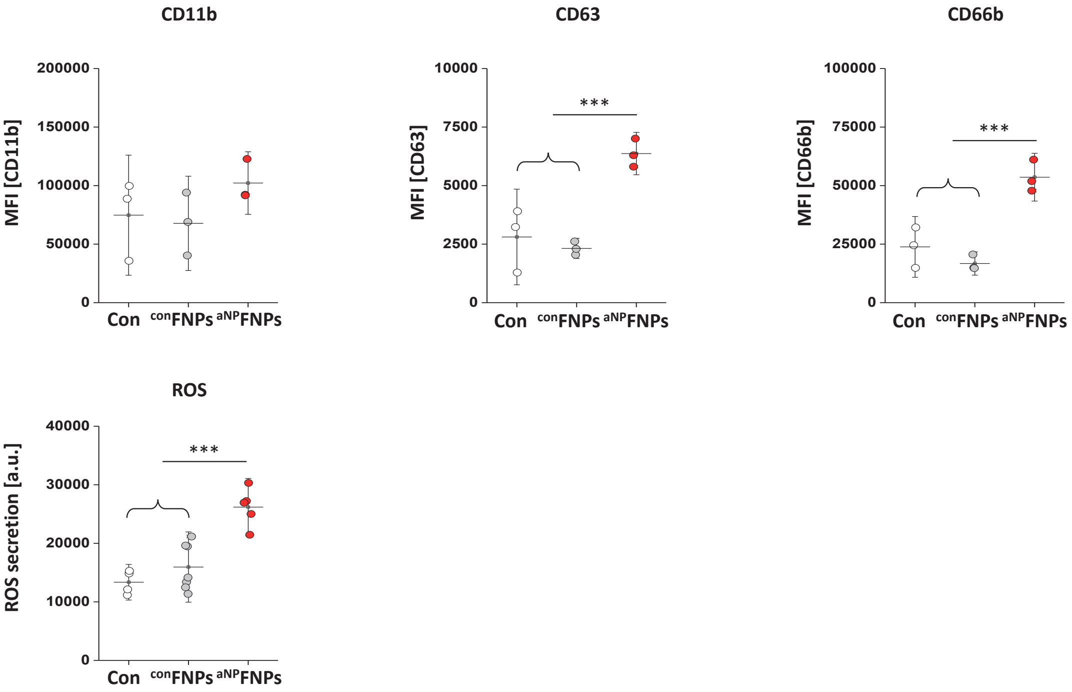

Figure S3: Response of human neutrophils/monocytes to ^{aNP}FNPs and free fMLP

Human whole blood was incubated for 1 hr at 37 °C under constant shaking with 10 µl/ml ^{aNP}FNP treatment or were left untreated. As positive control 0.1 µM fMLP was added. After 1 hr immune cells were isolated via erythrocyte lysis and cells were stained against CD11b for 20 min. at 4 °C. Finally, cells were washed once with 1 ml MACS buffer followed by determination of the CD11b surface expression *via* flow cytometric measurements.

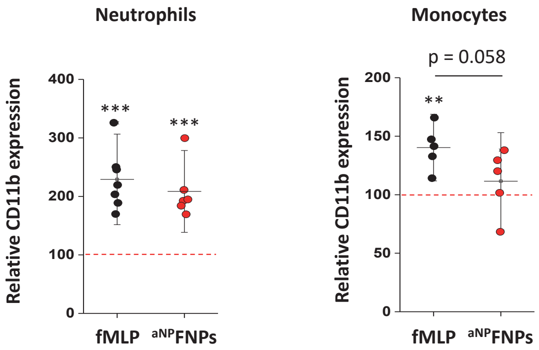

Figure S4: Impact of free EPICC and ^{iNP}FNPs on murine and human neutrophils

A + B) To investigate the impact of different EPICC concentrations on the ROS secretion by murine (A) and human (B) neutrophils, isolated neutrophils were stimulated with 100 nM PMA for 1 hr and in parallel incubated with different concentrations of the EPICC peptide. After 60 minutes of incubation, ROS secretion was determined via UPLC measurements and the values were normalized to neutrophils stimulated with PMA alone. The highest EPICC concentration lead to significant less secretion of ROS in mice and human neutrophils. **C)** Phagocytosis, expression of CD11b as well as migration was determined upon incubation of neutrophils with ^{iNP}FNPs. Values were normalized to untreated control cells. As shown we could not detect any influence of the ^{iNP}FNPs on phagocytosis, migration or early activation marker expression. **D)** To investigate if ^{iNP}FNPs are able to reduce ROS secretion by neutrophils, neutrophils were stimulated with PMA for 1 hr and in parallel incubated with ^{iNP}FNPs. Quantification shows that ^{iNP}FNPs can significantly reduce the ROS production of neutrophils. All data sets are mean value ± SD of n =3-5 (A), n = 3 (B), n = 5 (C) and n = 17 (D). * = p < 0.05, *** = p < 0.001 verified by one-way ANOVA (A+B) or Student's t-test (D).

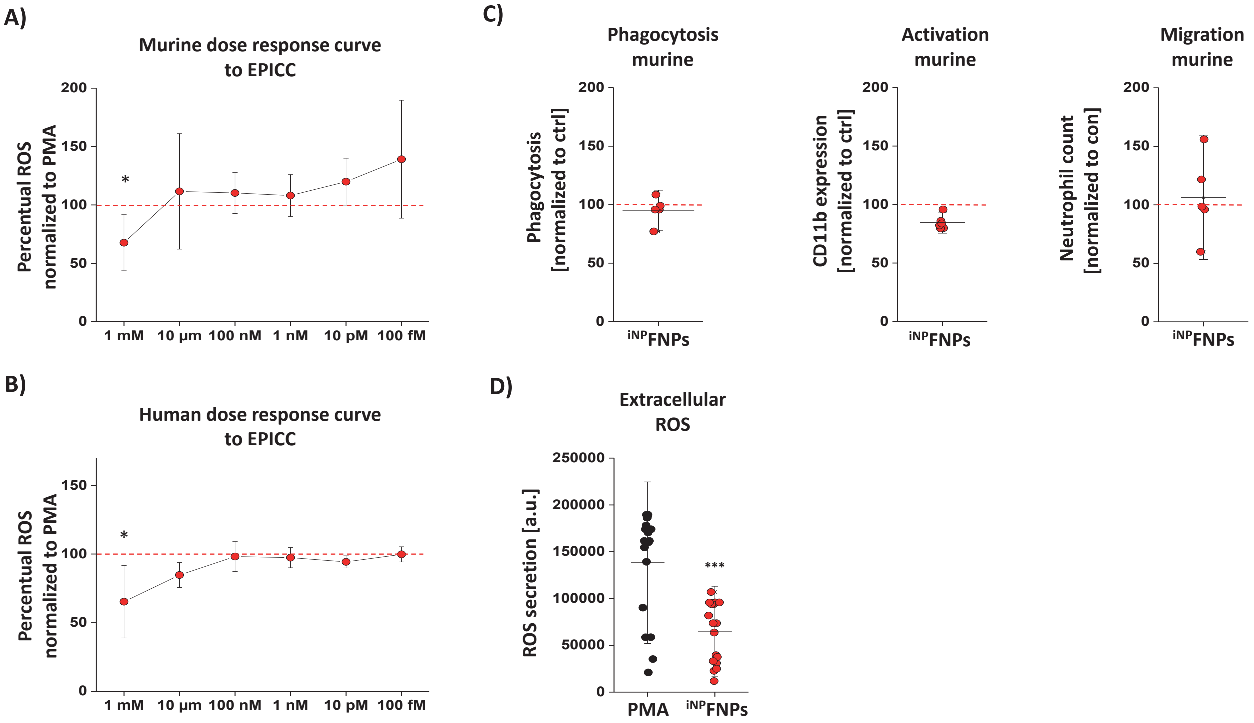

Table S1: Ingredients for FNP preparation

Abbreviations: ^{sNP}FNPs/^{bNP}FNPs = small/big neutrophil peptide-coupled fluorine-containing nanoparticles.

	^{sNP}FNPs	^{bNP}FNPs
Lipoid S75 (Lipoid AG, Ludwigshafen, Germany)	2.4% (w/w)	0.24% (w/w)
DSPE-PEG$_{2000}$ (Lipoid AG, Ludwigshafen, Germany)	0.45 mol%	0.45 mol%
DSPE-PEG$_{2000}$-maleimide (Avanti Polar Lipids, Alabaster, U.S.A.)	0.05 mol%	0.05 mol%
Atto647-DPPE (Atto-Tec, Siegen, Germany.)	0.025 mol%	0.025 mol%
Perfluoro-15-crown-5 ether (ABCR, Karlsruhe, Germany)	20% (w/w)	20% (w/w)
Phosphate glycerol buffer	up to 100%	up to 100%

Table S2: Scoring scheme for assessment of tissue damage in histologic gut sections

Abbreviations: LMM = lamina muscularis mucosae; SM = submucosa; M = mucosa; EIWS = entire intestinal wall section; LF = lymph follicle.

Points	Description
**I. Crypt depth**	
0: normal	Extends to LMM
1: slightly reduced	Extends to 2/3 M (affecting <10 % of EIWS)
2: strongly reduced	Extends to 2/3 M (affecting >10 % of EIWS)
3: not assessable	Complete loss of crypt structure
**II. Crypt structure**	
0: normal	Tubular, clearly demarked cellular structure
1: sporadically deformed	Affecting <10 % of EIWS
2: moderately deformed	Affecting 10 - 70 % of EIWS
3: strongly deformed	Affecting <70 % of EIWS
**III. Submucosa**	
0: normal	Tight, very thin, hard to demarcate
1: broadened	Increased connective tissue, loosened SM
2: detached	Broadened and detached from LM
**IV. Cellular destruction**	
0: none	Intact cellular structure, uncompromised tissue integrity
1: slightly increased	Increased detachment of surface epithelium
2: highly apoptotic	M (crypts) apoptotic, no clear demarcation of columnar epithelium, fibrous, disrupted cell surface
3: complete destruction	EIWS destructed
**V. Inflammation**	
0: none	LP very thin, no clear demarcation to LMM and crypt epithelium
1: little	LP fully surrounds basal parts of crypt epithelium
2: moderate	Increase of LP cells up to 10 % of total M or 1 distinct locus
3: strong	Increase of LP cells and broadened LP > 10 % of total M
**VI. Lymph follicles**	
0: none	no LF
1: <2	<2 LF
